# Genetical genomics of growth in a chicken model

**DOI:** 10.1186/s12864-018-4441-3

**Published:** 2018-01-23

**Authors:** Martin Johnsson, Rie Henriksen, Andrey Höglund, Jesper Fogelholm, Per Jensen, Dominic Wright

**Affiliations:** 1 The Roslin Institute and Royal (Dick) School of Veterinary Studies, University of Edinburgh, Easter Bush, EH25 9RG UK; 2 Department of Animal Breeding and Genetics, The Swedish University of Agricultural Sciences, Box 7023, 750 07 Uppsala, Sweden; 3 AVIAN Behavioural Genomics and Physiology Group, IFM Biology, Linköping University, 581 83 Linköping, Sweden

**Keywords:** Growth, Liver, QTL, Gene expression, eQTL, Genetical genomics

## Abstract

**Background:**

The genetics underlying body mass and growth are key to understanding a wide range of topics in biology, both evolutionary and developmental. Body mass and growth traits are affected by many genetic variants of small effect. This complicates genetic mapping of growth and body mass. Experimental intercrosses between individuals from divergent populations allows us to map naturally occurring genetic variants for selected traits, such as body mass by linkage mapping. By simultaneously measuring traits and intermediary molecular phenotypes, such as gene expression, one can use integrative genomics to search for potential causative genes.

**Results:**

In this study, we use linkage mapping approach to map growth traits (*N* = 471) and liver gene expression (*N* = 130) in an advanced intercross of wild Red Junglefowl and domestic White Leghorn layer chickens. We find 16 loci for growth traits, and 1463 loci for liver gene expression, as measured by microarrays. Of these, the genes *TRAK1*, *OSBPL8*, *YEATS4*, *CEP55*, and *PIP4K2B* are identified as strong candidates for growth loci in the chicken. We also show a high degree of sex-specific gene-regulation, with almost every gene expression locus exhibiting sex-interactions. Finally, several trans-regulatory hotspots were found, one of which coincides with a major growth locus.

**Conclusions:**

These findings not only serve to identify several strong candidates affecting growth, but also show how sex-specificity and local gene-regulation affect growth regulation in the chicken.

**Electronic supplementary material:**

The online version of this article (10.1186/s12864-018-4441-3) contains supplementary material, which is available to authorized users.

## Background

The molecular basis of variation in quantitative traits, such as body mass, is still very much an open question. Knowledge of how variation in body mass is affected by underlying genes and polymorphisms has been an area of intense study, and has ramifications for many diverse areas of biology, ranging from evolutionary theory, to human health, in the form of obesity risks, and to domestic animal production. For example, the genetic regulation of bodyweight in humans has proven to be complex but vital area of study [1], with over 13% of the worldwide population currently obese, whilst this prevalence has doubled since 1980, as estimated by the WHO. Various model species have been used for body weight analysis, ranging from *C.elegans* to *Drosophila* [2]. Domestication can be seen as a case-study in artificial selection for growth, and as such is an excellent tool in understanding the genetic basis of body mass regulation and control. Body mass has increased due to breeding in all major livestock species (reviewed by [3]). During domestication, and particularly the later phase of breed improvement, chickens have increased in body mass from between twofold to over fivefold as compared to their wild progenitor, the Red Junglefowl [4]. Similar to other species, chicken body mass is clearly polygenic, affected by variants at many loci. Body mass is a target of direct selection in chickens, particularly meat-type broilers [5]. It is also likely affected by correlated selection for egg production in layers, since there is genetic evidence of pleiotropy between traits [6], with body mass a strong determinant of egg size [7]. Finally, because body mass is likely related to fitness in the wild, wild and domestic chickens are likely to experience different selection pressure on body mass.

As is the case with all complex traits, there are very few known causal sequence variants that affect bodymass, yet some examples do exist. These indicate that diverse molecular processes can contribute to heritable body mass variation, some being of major-effect, far more being of small effect. There are Mendelian loss-of-function mutations that make animals small [8, 9]. For example, the sex-linked dwarfing locus in the chicken is caused by a growth hormone receptor mutation [10]. In mice, the *dwarf* locus is caused by a mutated *Pit-1* transcription factor, and loss of pituitary function, while the *little* locus is caused by mutation in the growth hormone-releasing hormone receptor [9]. There are also major quantitative trait loci (QTL) that have been resolved. In pigs, a regulatory variation affecting the insulin like growth factor 2 affects muscle growth and mass [11]. In mice, a major quantitative trait locus for body mass is caused by gene-regulatory variation at *Glypican 3* [12], and variation in the lipo-oxygenase gene *Alox5* affects gene expression and multiple metabolic traits [13]. Both of these loci appear to act through changes to liver gene expression. Genome-wide association studies of obesity in humans have isolated the *FTO*/*IRX3* locus, where it appears that a variant in an intron of the *FTO* gene affects body mass index by a regulatory effect on the neighbouring gene *IRX3* [14, 15].

To understand the mechanisms of chicken domestication, we need to know which genes have been affected by gene-regulatory variation in domestication. The genetic variants that affect traits must do so by means of affecting intermediary molecular phenotypes. A good way to get at the intermediary molecular traits is to genetically map gene expression levels, as measured by transcript abundance. This approach is called expression quantitative trait locus mapping or genetical genomics [16]. So far, it has revealed a plethora of gene-regulatory variation, with the strongest effects being local, putatively cis-regulatory, effects of genetic variants in or close to the gene itself [17, 18]. There is also a tendency for associations to cluster in the genome, into expression quantitative trait locus hotspots. This pattern is consistent with trans-regulatory changes affecting networks or pathways of genes [19] and with phenotypic buffering, where the phenotype is robust to genetic variation yet disruptions to key buffering loci can give rise to hotspots of QTL [20].

The genetical genomics approach allows us to integrate gene expression and trait mapping to find candidate quantitative trait genes that explain loci. Take the case where a regulatory genetic variant changes the expression of a gene, affecting a pathway that influences a phenotypic trait. This means that both the trait value and the gene expression level of the causative gene will be associated with the same genomic region. In this way, the overlap of QTL and expression quantitative trait loci (eQTL) can be used as a first step to filter potentially causative genes. For each overlap, gene expression is then correlated with the phenotype of the relevant growth QTL. In this way, putatively causal genes can be identified, with this pruning the number of candidates, and providing far stronger evidence of causation above basic overlap [7, 21, 22].

The liver is a key metabolic organ, involved in the metabolism of carbohydrates, proteins, and fats. It also both produces and breaks down hormones. Consequently, several studies have successfully used liver gene expression to investigate the genomics of metabolic traits [12, 13, 23, 24]. However, genetic variation in body mass could also act through other mechanisms, such as appetite regulation in the nervous system [25], or muscle growth in broiler chickens [26].

In this paper, we map QTL for body mass by linkage mapping in an advanced intercross of wild Red Junglefowl and domestic White Leghorn layer chickens. The use of an advanced intercross gives us improved mapping resolution for loci affecting body mass in chicken domestication. By allowing individuals to interbreed over multiple generations, the intercross accumulates recombinations, thus improving mapping resolution [27]. In the advanced intercross used for this study, we see approximately a four-fold increase in mapping resolution [28]. Given the key role of the liver as an effector of metabolism, we map eQTL for liver gene expression from a subset of 130 of the phenotyped animals. In this way, we find genes that have been affected by gene-regulatory variation under domestication. We integrate the genetic and gene expression evidence using association overlaps and linear models, with this then generating a final list of putatively causal genes affecting growth.

## Methods

### Mapping population

The intercross was started in the 1990s by crossing a Red Junglefowl rooster of Thai origin to three White Leghorn L13 layer hens [29]. The Leghorn line stemmed from a Scandinavian breeding experiment. The cross was previously expanded for mapping in the F_2_ generation. Since then, it has been maintained at a population size of approximately 100 individuals per generation. For the F_8_ generation (used in this study), chickens were raised in six batches. We weighed the chickens at hatch and days 8, 42, 112 and 212. We have body mass phenotypes for 566 birds at day 8 (270 males and 296 females), out of which 471 remained at day 212 (231 males and 240 females). Since a chick’s body mass at hatch is largely determined by the mass of the egg from which it hatched, which in turn is dependent on maternal factors [30, 31], maternal genetic effects may confound QTL for early growth and body mass. Therefore, we mapped early growth traits only as the difference between mass at day 42 and day 8. The full list of phenotypes is therefore weight at days 42, 112, and 212, and growth between days 8 and 42, 42 and 112, and 112 and 212. Chickens were culled by cervical neck dislocation followed by decapitation (as per the ethical permit). Animal handling was as per the ethical permit for the project.

### Quantitative trait locus mapping

All chickens (*n* = 566) were genotyped at 652 single nucleotide polymorphism markers by the Uppsala SNP&SEQ Technology Platform, using the Illumina Golden Gate assay. 551 markers were fully informative of parental origin, with the remainder being partially informative. The total map length was ~9268 cM, whilst the average marker spacing was ~16 cM. Additional file 1: Table S1 shows the genetic map with physical locations on the reference genome (version Galgal4). QTL for body mass at day 212 have been previously published in connection with brain proportion mapping [32].

We performed quantitative trait locus mapping using Haley-Knott regression [33] in the R/qtl package [34, 35]. Models included batch and sex as covariates, and genotype by sex interactions, where significant, were also included. Both additive and dominance effects were measured. Family effect was controlled for by including covariates of principal components based on global genotypes, with the first ten PCs checked against the phenotype to be tested, and all significant ones included in the model. We used both one-dimensional scans, detecting for single QTL, and two-dimensional scans for epistatic pairs of loci. In this way, we detect both marginal-effect and two-way epistatic loci, and then combined them in multiple-QTL models [36]. In this way, the multiple QTL model included covariates, main effects, sex interactions, and epistasis, starting with the most significant loci and working down for each trait. The power of the study (as calculated using the r/qtlDesign package [36]) was sufficient to give an 80% chance to detect a QTL of 4% effect size.

Permutation tests were carried out to find significance thresholds, using R/qtl, based on the classical and well established method as first outlined in [37, 38]. A 5% genome-wide LOD threshold was considered significant (LOD score ~4.3), whilst a 20% genome-wide threshold was considered as suggestive (LOD score ~3.7) [39]. Genomic confidence intervals were based on a 1.8 logarithm of odds (LOD) drop [40]. We compared the QTL with previously published growth loci from the F_2_ generation of the intercross, using the estimated physical locations in Animal QTLdb [41]. Epistatic interactions were also assessed using permutation thresholds generated using R/qtl, with a 20% suggestive and 5% significant genome-wide threshold again used. In the case of epistatic loci, the approximate average significance lod threshold for pairs of loci were as follows (as per the guidelines given in [36]): full model ~11, full versus one ~9, interactive ~7, additive ~7, additive versus one ~4. These are described in further depth in [36], but in brief these refer to the following: ‘Full’ refers to the lod threshold for the full two-locus model with additive and dominance effects included, ‘full vs 1’ is the threshold when comparing a two locus model as compared to a model with only one of the QTL pair fitted. ‘Lod.int’ gives the lod score threshold specifically for the interaction between the two QTL, ‘add’ gives the threshold for a two-locus model, but only considering additive effects, whilst ‘add versus 1’ gives the threshold for threshold for the additive two-locus model as compared to a single additive only QTL model.

### Liver RNA samples

A randomly chosen subset (130) of the F_8_ chickens that were used to map growth were also used for expression analysis. The subset used for gene expression were therefore not selected based on phenotype. Chickens were culled at 212 days of age. Liver samples were frozen in liquid nitrogen and stored at −80 °C. Frozen samples were homogenized with TRIzol (Qiagen) in a FastPrep MP-24 homogenizer (MP Biomedical), and total RNA was isolated according to the manufacturer’s protocol. We assayed RNA integrity on a Bioanalyzer (Agilent). The RNA integrity numbers ranged from 7.3 to 9.8 (mean 8.9).

### Gene expression microarrays

The eQTL sample consisted of 130 individuals from the intercross (67 males and 63 females). We made labelled cDNA with the Agilent Low Input Quick Amp Two Color Labeling kit. Agilent 8x60K arrays were used (with each array having 60 k probes and with 8 arrays per slide). Two different fluorophores were used (Cy3 and Cy5), enabling a total of 16 samples to be run per slide (2 samples per array and 8 arrays per slide). Arrays were hybridized according to the manufacturer’s protocol and scanned on a NimbleGen MS200 (Roche NimbleGen) scanner. We preprocessed array images with the Agilent Feature Extraction Software (version 12.0). These probe-level background-corrected values were then quantile-normalized, and summarized to probeset-level data by median polish using the preprocessCore R package (version 1.32.0 [42]). We used the ComBat method, implemented in the sva R package (version 3.18.0; [43]) to adjust for batch effects introduced by running multiple samples on the same slide.

Microarray probesets are based on Ensembl transcripts or RefSeq mRNA sequences, with 2–3 probes being used for each probeset, and summarized to one value per probeset. They were designed based on annotation from an older version of the chicken reference genome (WASHUC2.1/galGal3). To update the probesets, we first queried the Ensembl (version 85; [44]) database, through BioMart [45], for the current location of the genes underlying the probeset. In cases where the accession number had been retired, we used Blat [46] to align the probe sequences to Ensembl transcripts. When probe sequences aligned exclusively to transcripts from one Ensembl gene model, we annotated the probeset as belonging to that gene. This left us a total of 20,771 probesets, 16,360 of which were annotated to a genomic location.

### Expression quantitative trait locus mapping

We performed expression quantitative trait locus mapping with Haley Knott regression in R/qtl. We analysed local and distal eQTL separately, scanning only the 100 cM region around the gene (i.e. 50 cM upstream and 50 cM downstream), based on the genetic distance in the F_8_ map, for local eQTL. We estimated genetic effects coefficients for each eQTL using R/qtl’s fitqtl function. Each model was fitted with batch, sex and fluorophore (two different fluorophore colours were used in each microarray, Cy3 or Cy5) as covariates. We also fitted each eQTL model with and without a sex interaction, and compared the logarithm of the odds (LOD). In the cases where the sex interaction QTL has a LOD score greater than 1 this indicates that the individual sex * QTL interaction term in the QTL model has a ~*p* < 0.05 (the exact number can vary slightly from model to model), and the interaction was included in the final model. Permutation was once again used to generate significance thresholds for the local and trans eQTL. In the case of the local eQTL only the markers surrounding the gene in question were tested, with this reducing the multiple testing correction required. To account for the full number of eQTL probeset phenotypes (20771) and in the case of the trans eQTL the full genetic map, permutations were based on 100 randomly sub-sampled probesets (rather than single phenotypes). A similar method was used for the local eQTL, though in this case only the limited 100 cM region around each gene was used for extracting LOD scores (100 probesets were still considered simultaneously however). This generated a local threshold of LOD ~4.0 and a trans LOD threshold of ~8.0. The power of the study (as calculated using the r/qtlDesign package [36]) was sufficient to give an 80% chance to detect a QTL of 16% effect size.

For each phenotypic quantitative trait locus, we found the overlapping local eQTL, and tested each of them for an association between the growth trait in question and gene expression. We used linear models with the growth trait value as response variable, and the gene expression value as predictor, with fluorophore (Cy3 or Cy5), and batch as covariates.

Gene Ontology annotation was downloaded from Ensembl (version 85) through BioMart.

### Expression quantitative trait locus hotspots

We calculated the number of expression quantitative trait locus confidence intervals covering every part of the genome with the GenomicRanges R package [47], and compared it to the coverage of intervals in simulations where the intervals were placed at random. To avoid the risk of spurious hotspots driven by extreme individuals and skewed genotypes, we filtered out potential trans-eQTL where there were ten or fewer individuals of any genotype class. To generate a null distribution of eQTL, we simulated placing the distal eQTL confidence intervals randomly on a 1 Gb interval (the size of the sequenced autosomal chicken genome), and found the highest coverage in each simulation.

We also tested to see whether any trans-eQTL hotspots were potentially controlled by any of the local eQTL that overlapped the trans hotspot location. This was performed using a conditional eQTL model. First, we identified any local eQTL that overlapped the hotspot, and then performed a test of correlation between the expression levels of the respective cis and trans eQTL phenotypes. We used the residual gene expression value from a model that included sex, batch and fluorophore. Finally, we repeated the trans eQTL detection model (i.e. how genotype modifies gene expression), but now also included the expression of the local eQTL gene as a covariate in the trans-eQTL model. If the cis eQTL is a causal to the trans eQTL hotspot the inclusion of the cis eQTL as a covariate should reduce the variance explained by the genotypic effect (i.e they both explain the same variance component), and hence the strength of the trans eQTL should drop. We considered the local eQTL as a potential mediator of the trans-association if 1) there was a significant association between the expression values, and 2) the logarithm of the odds of the trans-eQTL was reduced by more than half when the local gene was included as a covariate in the trans-eQTL model. We used the circlize R package [48] for the circular plot of hotspots, and the igraph R package [49] for network plots. It must also be noted that such loci are only putative – rather than the trans eQTL being downstream targets of the local gene, it is impossible to rule out that the causal element has both local and trans regulatory properties.

### Selective sweep overlap

We compared the QTL with selective sweep signals detected by [50] by searching for overlaps between layer and all domestic sweep regions and quantitative trait locus support intervals. We used WASHUC2.1/galGal3 genome coordinates, because the sweep dataset was based on that version of the reference genome. We generated an empirical null distribution by uniformly random placement of sweeps and counting the overlaps.

## Results

### QTL for body mass

**Fig. 1:**
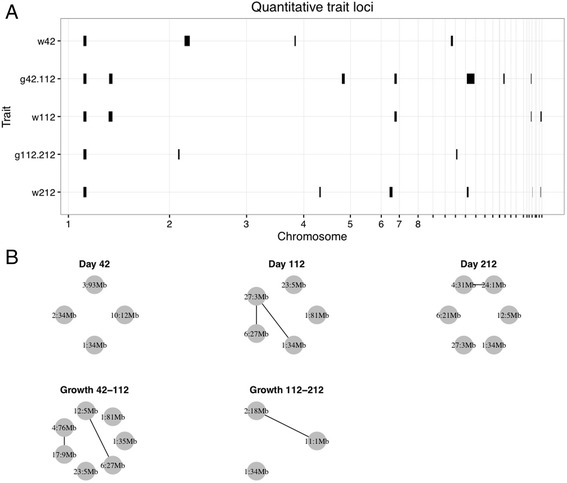
**a** Genomic confidence intervals of the QTL. The horizontal axis shows physical locations along the chicken genome (chromosomes 1 to 28). The red lines indicate QTL overlap between at least two traits. **b** Epistatic networks for body mass and growth loci. Nodes represent loci, labelled by their chromosome and physical location, and edges pairwise epistatic interactions between them

We found a total of four loci for body mass at 42 days, five for body mass at 112 days and six for body mass at 212 days. We also found seven loci for growth between day 42 and 112, and three loci for growth between day 112 and 212. In total, this amounts to 16 genomic regions associated with body mass or growth (Table 1, Fig. 1a). There was some epistasis evident in the cross, in the form of six two-locus interactions (Fig. 1b).

**Table 1:** QTL with estimates and position

Chr	cM	Mb	LOD	Trait	a	SE	d	SE	a_by_sex	SE	d_by_sex	SE	Interactions	Interval start (Mb)	Interval end (Mb)	Variance explained (%)
1	509	34	27.0	w42	19.9	2.1	9.6	2.7						28.9	34.7	4.0
1	510	34	5.5	g112.212	31.4	6.4	−4.8	8.0						28.9	34.7	2.0
1	510	34	42.4	w112	83.3	13.3	64.3	16.6	−34.9	13.0	0.6	17.6	1@510.0:27@68.2	28.9	34.7	9.0
1	510	34	43.6	w212	159.8	13.1	15.6	17.1	−75.4	16.8	35.9	23.2		28.9	34.7	8.4
1	512	35	35.3	g42.112	85.3	7.9	14.2	10.2	−47.4	10.0	20.1	14.0		28.9	34.7	7.0
1	1138	81	4.3	w112	−25.6	6.0	−7.0	8.0						77.6	85.0	0.8
1	1137	81	5.1	g42.112	−23.1	5.2	−9.0	6.9						78.6	85.0	0.9
2	158	18	9.4	g112.212	1.2	7.1	−6.5	9.4					11@4.0:2@158.0	16.7	19.3	3.4
2	255	34	5.2	w42	12.6	3.8	−27.7	6.8	−8.8	5.3	29.1	9.3		29.0	39.1	0.7
3	631	93	7.5	w42	−19.8	4.5	−7.2	6.8	8.7	5.7	−6.7	8.9		92.6	95.1	1.0
4	265	31	8.6	w212	7.8	9.8	19.0	12.3					4@265.0:24@14.1	30.2	32.9	1.4
4	493	76	11.7	g42.112	−9.2	7.6	−7.3	9.7	30.7	9.3	−28.2	13.4	4@493.0:17@169.0	73.8	79.3	2.1
6	207	21	5.0	w212	26.2	8.2	29.3	11.1						16.0	21.6	0.8
6	259	27	13.8	g42.112	15.5	5.5	53.4	7.6					6@259.0:12@66.0	25.5	29.9	2.5
6	258.7	27	9.0	w112	26.6	13.1	37.3	14.5					27@68.2:6@258.7	25.5	29.9	1.6
10	177	12	4.4	w42	8.8	2.4	8.2	2.9						11.1	15.1	0.6
11	4	1	9.2	g112.212	−5.1	6.9	−16.6	9.1					11@4.0:2@158.0	1.3	3.8	3.4
12	66	5	8.6	g42.112	12.2	7.2	−35.5	10.0					6@259.0:12@66.0	2.7	17.4	1.5
12	64	5	4.4	w212	34.7	10.2	−19.8	14.4						2.7	5.9	0.7
17	169	9	8.3	g42.112	20.6	6.0	−25.5	8.7					4@493.0:17@169.0	7.2	9.5	1.5
23	147	5	4.8	w112	−31.0	7.0	7.4	11.1						3.5	4.7	0.9
23	154.9	5	3.8	g42.112	−20.4	5.1	0.8	6.8						3.5	4.7	0.7
24	14.1	1	9.5	w212	1.1	9.0	−38.1	10.9					4@265.0:24@14.1	1.1	1.5	1.5
27	68.1	3	4.2	w212	37.8	11.1	−1.7	13.3						2.4	3.4	0.7
27	68.2	3	9.6	w112	43.6	15.0	19.2	16.1					1@510.0:27@68.2 27@68.2:6@258.7	2.4	4.7	1.8

Columns indicate the trait, the location of the QTL (in cM and Mb), the LOD score for the QTL, the additive and dominance estimates with standard errors, the coefficients for additive and dominance by sex interactions with standard errors, epistatic pairs that the QTL contribute to, the limits of the QTL confidence interval (in Mb), and the variance explained

The loci for body mass at earlier and later time points suggest a different architecture for early growth and adult body mass. This cross has a major body mass locus on chromosome 1, previously named *growth1*, which was the only locus that is detected at all ages. Except for *growth1*, there was no overlap between body mass loci at day 42 and day 112, and only one more overlapping locus (on chromosome 27) between day 112 and day 212. In comparison with previously published QTL from the F_2_ generation of the intercross [4], we replicated four QTL regions on chromosomes 1, 2, 12 and 27 (Additional file 2: Figure S1). There was also another overlapping locus on chromosome 6 with a previously published reanalysis of the F_2_ data [51]. We also overlapped the detected QTL with the selective sweeps due to domestication and improvement detected by [50] (overlaps given in Additional file 1: Table S1). Multiple overlaps were observed between selective sweeps and QTL, although there was no significant enrichment of sweeps observed in QTL regions (i.e the numbers were not significantly different from those that would be observed by chance). The most sweeps appeared to be more apparent in the growth phenotypes. For example, g112–212 (growth between 112 and 212 days) had a total of 11 sweeps overlapping 3 QTL (5 sweeps seen in all domestic birds and 6 sweeps observed in layer birds), whilst g42–112 (growth between 42 and 112 days) had 11 sweeps overlapping 7 QTL regions (5 ‘all domestic’ sweeps and 6 layer-specific sweeps). In comparison, weight at 212 days only had 5 sweeps overlapping 6 QTL regions. This could indicate that selection is acting principally on growth (particularly early to intermediate growth stages) rather than final adult weight.

### Gene expression quantitative trait loci in liver

**Fig. 2:**
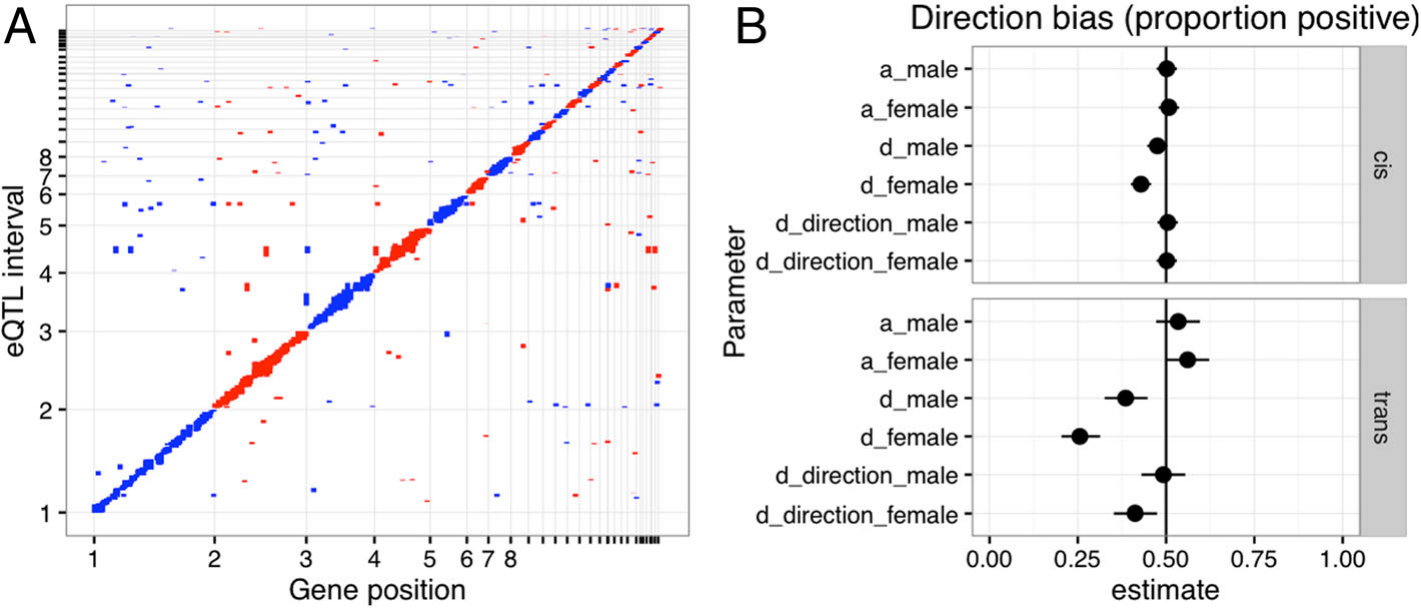
**a** eQTL local-distal plot. Plot of the genomic position of the expression quantitative trait locus confidence intervals (vertical axis) and their respective gene (horizontal axis). The diagonal represents local eQTL, mapping to the location of the gene itself. **b** Proportions of positive and negative genetic effects

We found 1463 eQTL, 1201 local and 262 distal trans-acting (Fig. 2a, Additional file 3: Table S2). Evidence of a sex x QTL interaction was seen in 1077 local (90%) and 260 (99%) distal (trans) loci.

The additive effects of the eQTL appear to have no bias in the direction of effect. Approximately as many loci were associated with a higher expression in the Junglefowl allele as the White Leghorn allele, in both males and females for both local and distal loci (Fig. 2b). Given that we have no reason to expect biased genotypic effects, this result is as expected. However, when it comes to the dominance effects, local and distal loci seem to differ. The local eQTL in males again appeared unbiased. However, the majority of female local eQTL and trans eQTL in both sexes had a negative dominance effect, meaning that the heterozygote expression was lower than expected by the mid-parental value. We also investigated the direction of dominance in terms of bias of the heterozygote value towards the Red Junglefowl or White Leghorn homozygote, and found no evidence of a directional bias in any sex. The strongest local eQTL include genes that likely effect liver function. We found eQTL affecting mitochondrial NADH dehydrogenases *NDUFA8* (LOD = 40) and *NDUFS6* (LOD = 16), *transforming growth factor beta 2* (*TGFB2*, LOD =18), and redox enzymes *thioredoxin* (LOD = 15) and *thioredoxin reductase 3* (LOD = 23). We also found a female-specific locus for *beta-carotene oxygenase 2* (*BCO2*, LOD = 21), which causes the yellow-skin phenotype in domestic chickens (Additional file 4: Figure S2). The most common Gene Ontology Biological Process terms among genes with eQTL were related to transcription, signal transduction, transport, and protein phosphorylation (Additional file 5: Table S3).

### Genetical genomics search for candidate quantitative trait genes

**Fig. 3:**
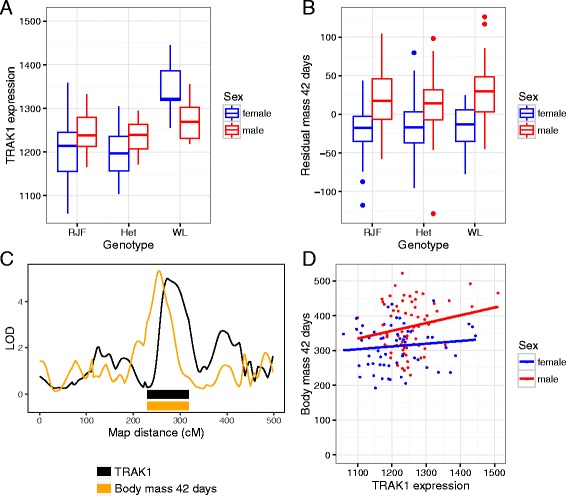
Plots of individual phenotypes versus their genotype (**a**, **b**), Logarithm of the odds curves (**c**), and scatterplots of body mass versus gene expression for *TRAK1* for body mass at 42 days (**d**)

By overlapping phenotypic and gene eQTL, we searched for candidates for body mass at different ages (Table 2; Fig. 3; Additional file 6: Figure S4; Additional file 7: Figure S5 and Additional file 8: Figure S6). We considered a gene a candidate quantitative trait gene if: 1) It had a local expression quantitative locus overlapping the confidence interval of the phenotypic locus, and 2) the expression level of the gene was also associated with the trait, allowing for sex-interaction and adjusting for technical covariates. In total five candidate genes were identified using this approach. *Trafficking protein, kinesin binding 1* is a candidate for body mass at 42 days on chromosome 2 (*TRAK1*; ENSGALG00000011938, LOD = 5, correlation *P* = 0.003). *YEATS domain-containing protein 4* (*YEATS4*; ENSGALG00000029135, LOD = 5.3, correlation *P* = 0.006) and *oxysterol binding protein like 8* (*OSBPL8*; ENSGALG00000010246, LOD = 4.4, correlation *P* = 0.0035) are candidates for body mass at 42 days on chromosome 1, at *growth1*. Given its apparent female-specific effect, *YEATS4* seems a less likely candidate than *OSBPL8* for *growth1*, which has a clear effect on both sexes. However, *growth1* could also be made up of multiple linked loci. There was a second LOD score peak for body mass at day 42 and 112 (Additional file 9: Figure S7). If so, the location of *OSBPL8* is suggestive of a role in this second locus. *Centrosomal protein 55* (*CEP55*; ENSGALG00000006639) is a candidate for body mass at 112 days on chromosome 6. On chromosome 27, *Phosphatidylinositol-5-phosphate 4-kinase type-2 beta* (*PIP4K2B*; ENSGALG00000001610) is a candidate for the adult body mass locus. The localization of the expression quantitative trait locus, however, is imprecise for *PIP4K2B,* with a peak relatively far from the peak of the phenotypic locus.

**Table 2:** Candidate genes from expression quantitative trait locus mapping

Gene name	Chr	Start	Trait	LOD	p-value	Interval start	Interval end	Ensembl gene ID
YEATS4	1	35.4	w42	5.3	0.0060	33.6	44.1	ENSGALG00000029135
OSBPL8	1	38.0	w42	4.4	0.0035	33.6	45.7	ENSGALG00000010246
TRAK1	2	44.3	w42	5.0	0.0026	29.0	39.1	ENSGALG00000011938
CEP55	6	20.0	w112	4.7	0.0042	21.6	25.5	ENSGALG00000006639
PIP4K2B	27	4.0	w212	5.7	0.0017	1.2	4.7	ENSGALG00000001610

Columns indicate the name of the gene, its chromosome and location (in Mb), the LOD score for the eQTL, *p*-value for the regression between gene expression level and trait value, the locations of the eQTL confidence interval (in Mb), and the Ensembl gene ID

### Trans-regulatory hotspots

**Fig. 4:**
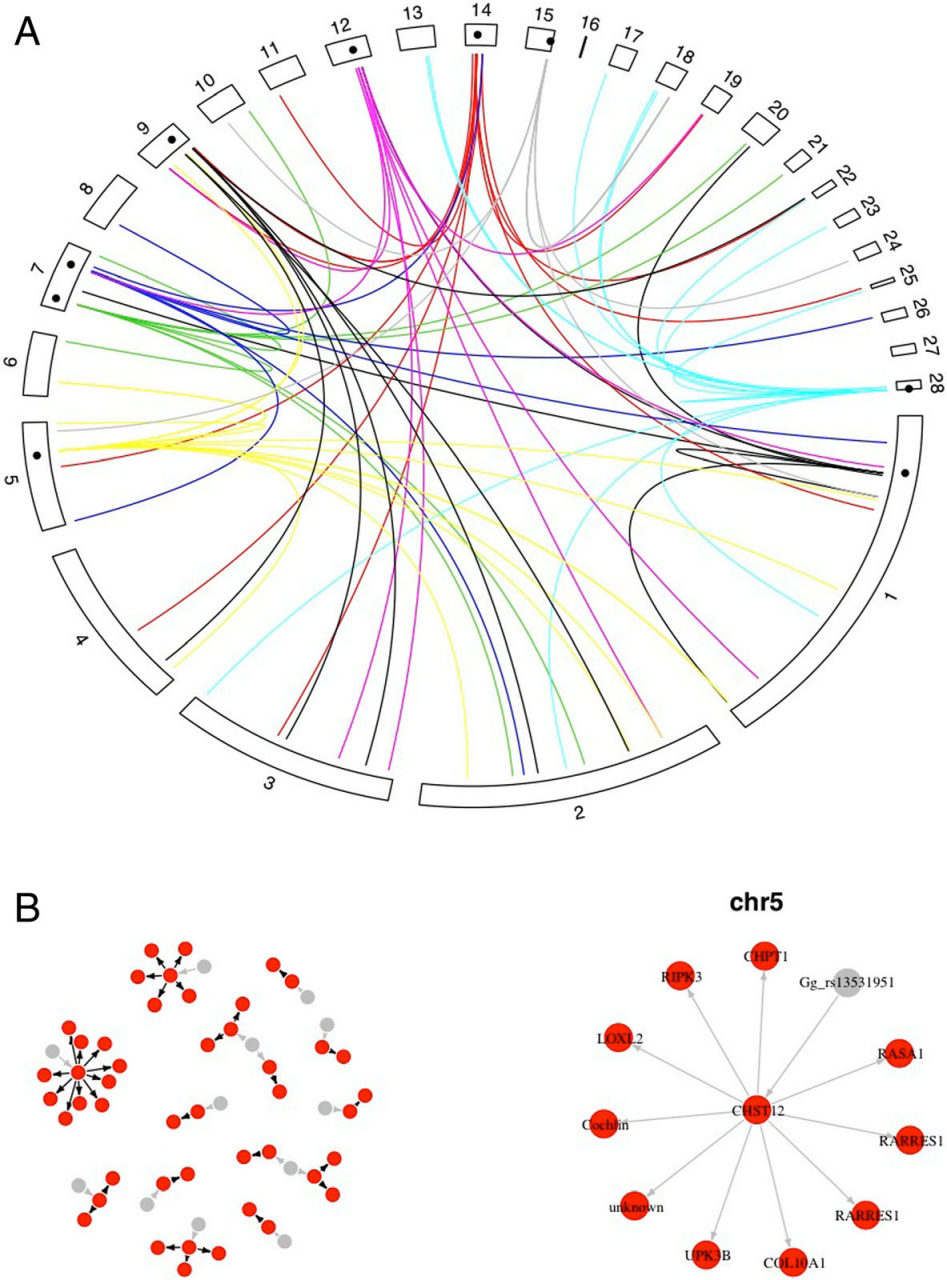
**a** Circular genome plot of putative trans-eQTL hotspots. The points show the location of hotpots on the genome, and the arcs show trans-eQTL associated with the hotspot. Each hotspot has its own colour. **b** eQTL network plots with the chromosome 5 hotspot zoomed in. Grey nodes represent markers associated with genes. Red nodes represent probesets. The edges represent potential regulatory relationships from a local eQTL to distal trans-eQTL

We found nine putative trans-regulatory hotspots, where more expression quantitative trait locus mapped than would be expected by chance (Fig. 4a, Additional file 10: Table S5). Two of them, on chromosomes 1 and 12, overlapped phenotypic QTL: the chromosome 1 hotspot overlapped *growth1*, and the chromosome 12 hotspot overlapped a locus for growth from day 42 to 112. We used local eQTL overlapping trans-acting loci to search for genes that may regulate multiple genes (Fig. 4b). The resulting network only included 32 of the distal, putatively-trans acting loci. In one case, the hotspot on chromosome 5, we found a regulatory candidate that may explain the associations (Fig. 4c). At the chromosome 5 hotspot, the analysis suggested that a gene-regulatory variant affecting *carbohydrate sulfotransferase 12* (*CHST12*; ENSGALG00000004276) had downstream effects on *collagen 10A1* (*COAA1*; ENSGALG00000014965), *retinoic acid receptor responder protein 1* (*RARRES1*; ENSGALG00000009594, represented by two probesets), *lysyl oxidase homolog 2* (*LOXL2*; ENSGALG00000000402), *RAS p21 protein activator (GTPase activating protein) 1* (*RASA1*; ENSGALG00000017706), and an uncharacterized gene (ENSGALG00000001885). This hotspot appeared to be female-specific (Additional file 4: Figure S2; Additional file 11: Figure S3). We did not try to infer the connections between the trans eQTL as there may be more complicated direct and indirect regulatory relationships between them.

### Correlations between gene expression and growth traits

In addition to testing the overlapping QTL/eQTL genes, a search for genes whose expression is associated with growth and body mass regardless of the presence of an eQTL was also conducted. To this end, we used the same linear model as for the positional candidate genes above, but applied it across the whole array using a permutation test. In this way, we found 40 probesets associated with traits (Additional file 12: Table S4). The list includes regulatory genes such as *SOX5* (*ENSGALG00000013204, P = 7 × 10*^*−5*^), and *BDNF* (*ENSGALG00000012163, P = 2 × 10*^*−5*^). The list does not overlap with the candidate genes found for the phenotypic loci. However, two of these candidates had local eQTL. One of them, *LOC776181 similar to receptor for egg jelly-like protein* (*ENSGALG00000014240*, *P = 1 × 10*^*−4*^) also overlapped a phenotypic quantitative trait locus for the same trait, namely body mass at 112 days, and in this way can also be considered to be a putative candidate gene.

## Discussion

In this paper, we perform quantitative trait locus mapping of growth traits, and expression quantitative trait locus of liver gene expression in an advanced intercross of wild Red Junglefowl and domestic White Leghorn layer chickens. We find that the genetic architecture of body mass is specific to different ages, with little overlap between QTL for early growth and adult body mass. The only locus detectable at every age is the major growth locus, *growth1*, which explains around 9% of the variance in weight. One caveat with this is that smaller, undetected loci may well be shared between early and adult growth traits but have not been detected in this study. By measuring liver gene expression in a subset of the cross (130 individuals), we detected 1463 eQTL.

Using an integrative genomics approach we identified candidate genes for some of the loci for body mass at 42, 112 and 212 days. Two of the candidate genes have known connections to metabolism or cell proliferation. *TRAK1* is involved in mitochondrial transport and attachment to microtubules [52, 53]. In broiler chickens, differences in mitochondrial function are associated with feed efficiency [54, 55]. This suggests that genetic effects on the expression of mitochondrial proteins could affect growth in chickens. *OSBPL8* binds oxysterols, and changing its expression in transgenic mice, altering lipid levels in liver and blood [56]. The other candidate for this locus, *YEATS4* is involved in gene regulation and cell proliferation [57]. It is also located close to a genome-wide association signal for human height that occurs in the neighbouring gene *FRS2* [58, 59]. *CEP55* encodes a microtubule-associated protein involved in cell division [60] and is required for embryonic development in zebrafish [61]. *PIP4K2B* is an enzyme that phosphorylates phosphatidylinositol 5-phosphate. The product is involved in regulation of cell proliferation, and overexpression of *PIP4K2B* can be contribute to cancer [62].

The quantitative trait locus analysis of body mass replicates five out of the 13 loci from the F_2_ generation of the intercross [4, 63]. As opposed to the marginal effect loci, the patterns of epistasis found in the F_2_ generation do not replicate. For example, we detect again loci on chromosome 1, 6, 12 and 27 for adult body mass from the F_2_ reanalysis [51]. In the previous study, these loci were all involved in at least one epistatic interaction, while we find no epistatic interactions between any of them. There are several reasons why epistatic effects in an F_2_ cross may not replicate in an advanced intercross. Firstly, the previous analyses used different software, and slightly different phenotypes (in that phenotypes were recorded at slightly different ages, though always within a few days of each other between the two studies), though these are both relatively minor differences. Secondly, the longer linkage blocks in an F_2_ intercross cause lower resolution, and what one detects may be synthetic associations made up of several linked loci. As recombination reduces linkage over generations, the apparent epistatic patterns may change. Another reason for the lack of detected epistasis may be the unbalanced allele frequencies of the advanced intercross. Allele frequencies have changed by drift over the generations, which make genotype frequencies deviate from the near perfect 1:2:1 Mendelian ratios of an F_2_. This makes some multilocus genotypes rare, reducing the power to detect epistasis. Regardless, our results do not support a major effect for epistasis in chicken growth under domestication. We find some epistatic pairs, but most loci lack significant epistatic effects, and only one locus is involved in more than one interaction. Finally, there was no enrichment of selective sweeps, previously detected in chicken domestication [50], in the growth and body mass QTL regions. However, despite there being no significant enrichment, multiple selective sweep regions were nevertheless detected within the various QTL. These may very well reflect genuine selection signals on growth or weight phenotypes. The clearest way to prove or disprove recent selection is the cause of these QTL is to identify the underlying causal variant(s) for each QTL and determine their position relative to these sweeps, though this is by no means a trivial task. Intriguingly, if these sweeps are causal to the observed QTL, this would indicate that selection has been principally acting on growth rather than final weight.

Most of the eQTL are local. This agrees with the general results of expression quantitative trait studies, from various organisms, that genetic variation in gene expression is abundant, and often local. However, there is also the general issue of the higher significance threshold for trans eQTL making them harder to detect, and potentially downwardly biasing their detection. When it comes to distal, putatively trans-acting eQTL, we find nine potential trans hotspots. Further investigation would be needed to know whether these are genuine trans-regulatory hotspots and what traits they may participate in. Interestingly, most of the eQTL exhibit sex-interactions. This indicates that males and females potentially have distinct architectures for liver-specific gene expression, or at the very least the size of the effect of many of the eQTL varies between the sexes. Given the liver’s role in energy balance, and the extreme size difference between male and female chickens, this may not be unusual. For example, extensive sexual dimorphism in liver gene expression has been observed in the mouse [64]. This study found that ~72% of the genes expressed in mouse liver were sexually dimorphic in expression (in comparison ~13% of genes were sexually dimorphic in the brain). Given such sex-differences seem to be present in the liver transcriptome, sex x genotype interactions (when the eQTL allele has a greater effect in one sex) are less surprising. This consistent relationship between the liver transcriptome and sex bias seen in both chickens and mice could potentially reflect the degree of sexual dimorphism present in body size, with the chickens increased size sexual dimorphism (as compared to the mouse) being reflected in the slightly greater liver transcriptome sex bias, though this remains to be tested further. Sex interaction may also be exaggerated because of the lower power to detect sex-specific eQTL. In particular, because of the signal to noise ratio of the microarrays it may be harder to detect an expression quantitative trait locus in the sex with lower expression (in these instances, usually females). This sex interaction does raise a caveat regarding the candidate genes that were identified, as the eQTL candidates interacted between the sexes, whereas the bodyweight QTL for the most part did not. This could indicate that different genes underlie the same QTL in males and females, or that the lower power in the eQTL scan makes identifying the weaker sex-effect more problematic.

## Conclusions

In conclusion, we detected 16 loci affecting growth traits in chicken domestication and 1463 eQTL affecting genes that may change liver function. We also highlight six potential candidates genes for affecting body mass in the chicken.

## Additional files

Additional file 1: Table S1. Number of overlaps between quantitative trait locus regions and selective sweep regions from [50]. (CSV 426 bytes)

Additional file 2: Figure S1. QTL from this study and previous F_2_ analysis [4] and reanalysis [51] based on physical locations in Animal QTLdb. Since the entries from the reanalysis lack confidence intervals, points indicate the loci. (PDF 5 kb)

Additional file 3: Table S2. All eQTL. (CSV 124 kb)

Additional file 4: **Figure S2** Female-specific expression quantitative trait locus for BCO2, showing gene expression level as a function of genotype. (PDF 6 kb)

Additional file 5: Table S3. Most common Gene Ontology Biological process terms among eQTL. (XLSX 8 kb)

Additional file 6: Figure S4. *YEATS4* and *OSBPL8* candidate plots. (PDF 19 kb)

Additional file 7: Figure S5. *CEP55* candidate plots. (PDF 11 kb)

Additional file 8: Figure S6. *PIP42KB* candidate plots. (PDF 10 kb)

Additional file 9: Figure S7. Logarithm of odds curves for the region of chromosome 1 containing the two significant QTL, and a potential second peak, after *growth1,* which may be a second, imperfectly resolved QTL. The dashed lines indicate genome-wide significance thresholds for the respective trait. (PDF 7 kb)

Additional file 10: Table S5. Trans-expression quantitative trait locus hotspots. (XLSX 35 kb)

Additional file 11: Figure S3. Plots of gene expression versus genotype for eQTL of the chromosome 5 hotspot. (PDF 22 kb)

Additional file 12: Table S4. Genes with expression levels associated with growth traits. (CSV 2 kb)

Additional file 13: Genotype and phenotype data underlying quantitative trait locus mapping analysis in R/qtl format. (CSV 1191 kb)

Additional file 14: Genotype and phenotype data underlying expression quantitative trait locus mapping analysis in R/qtl format. (CSV 39860 kb)
