# Supplementary figures and images for: Genetical genomics of growth in a chicken model
Source: BMC Genomics. 2018 Jan 23;19:72. doi: 10.1186/s12864-018-4441-3 (PMC5782384; doi:10.1186/s12864-018-4441-3)

# Quantitative trait loci

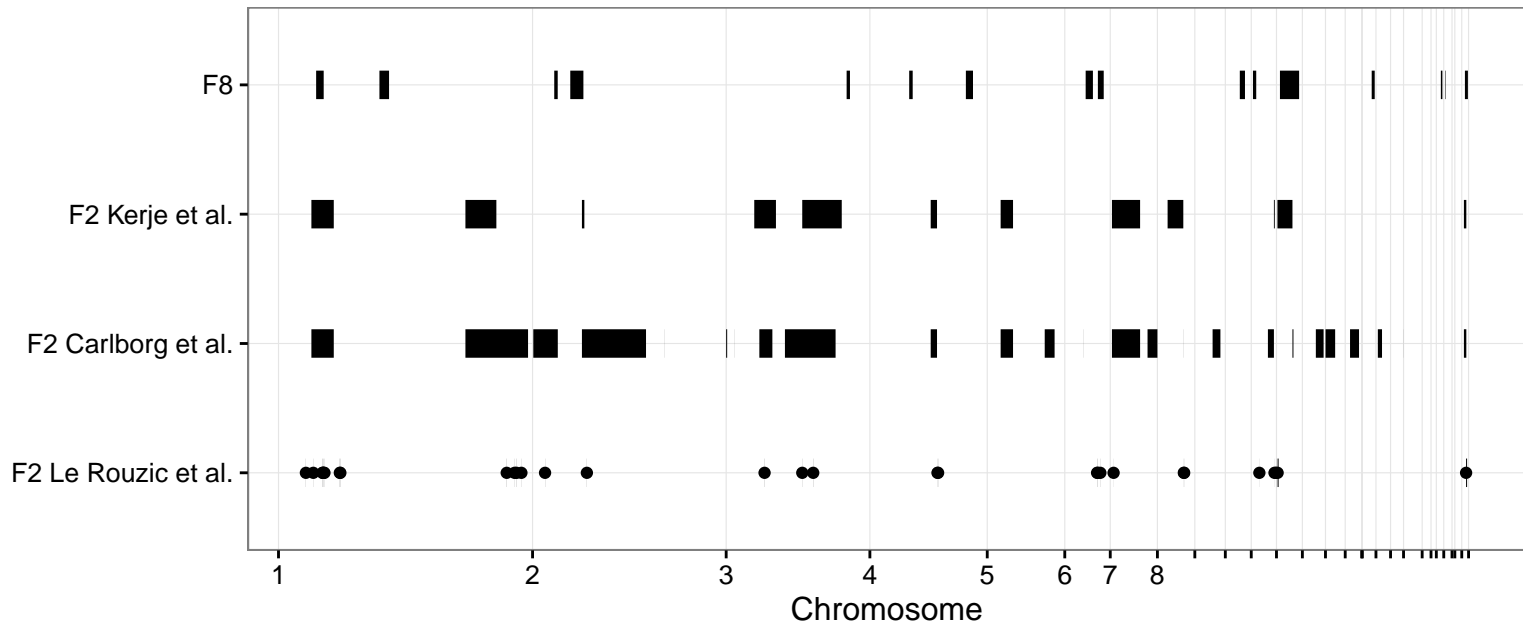

Supplement: Supplementary file 2 — QTL from this study and previous F2 analysis [4] and reanalysis [51] based on physical locations in Animal QTLdb. Since the entries from the reanalysis lack confidence intervals, points indicate the loci. (PDF 5 kb) [file 12864_2018_4441_MOESM2_ESM.pdf]

BCO2

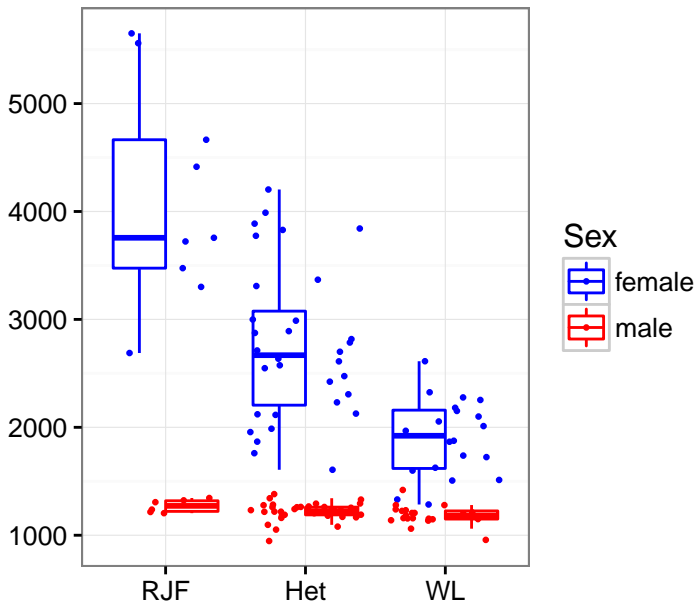

Supplement: Supplementary file 4 — Figure S2 Female-specific expression quantitative trait locus for BCO2, showing gene expression level as a function of genotype. (PDF 6 kb) [file 12864_2018_4441_MOESM4_ESM.pdf]

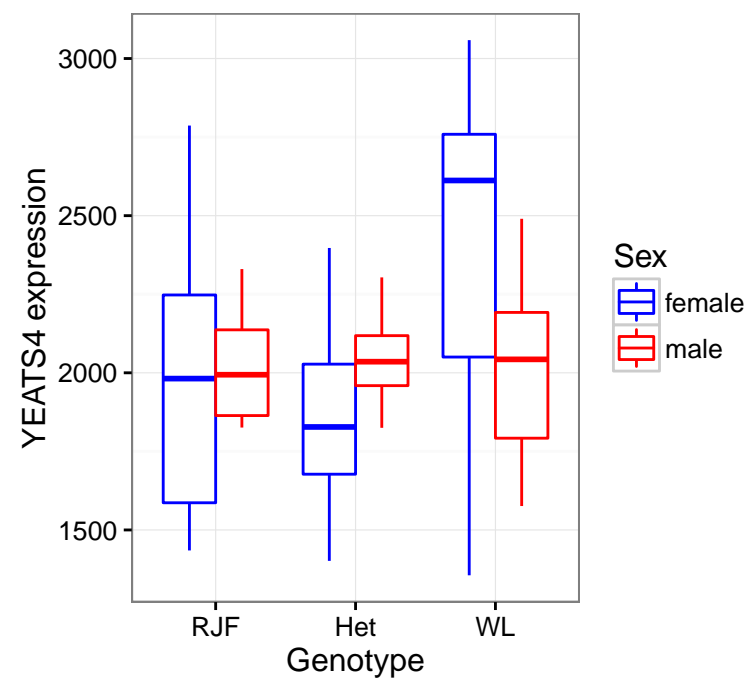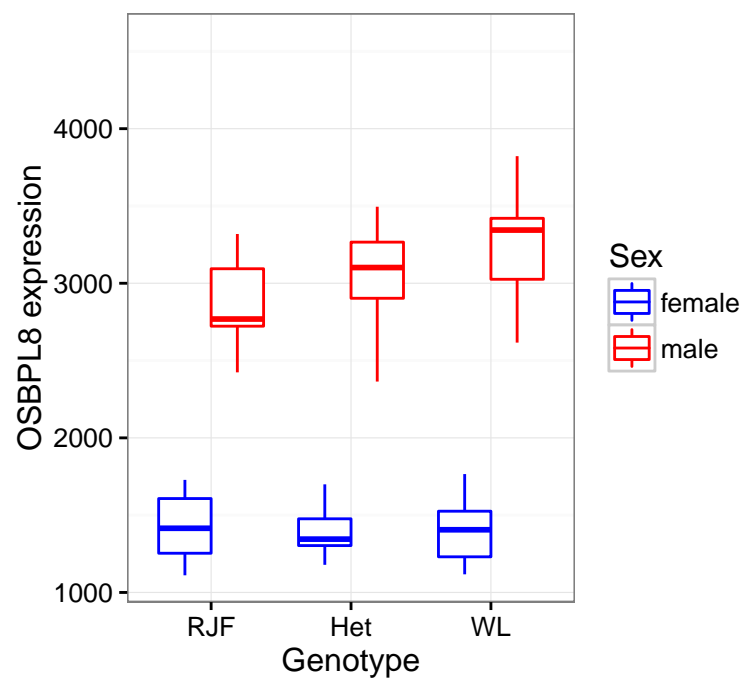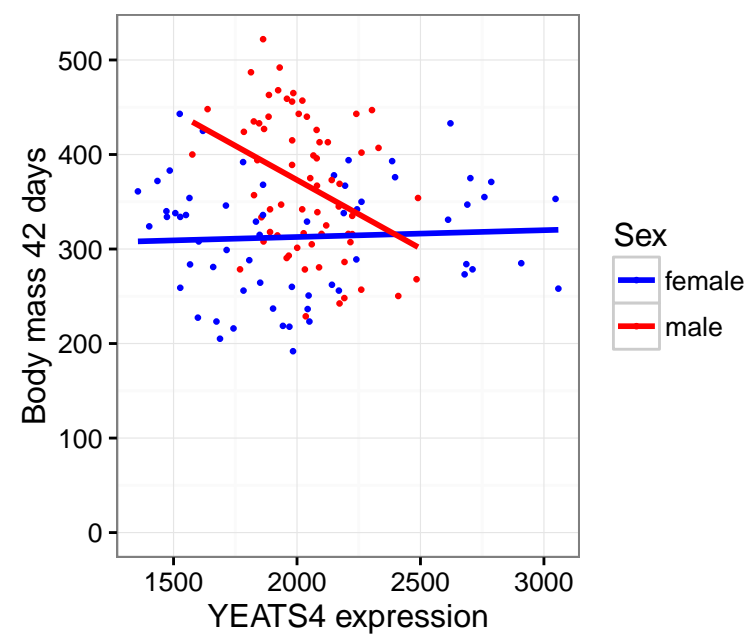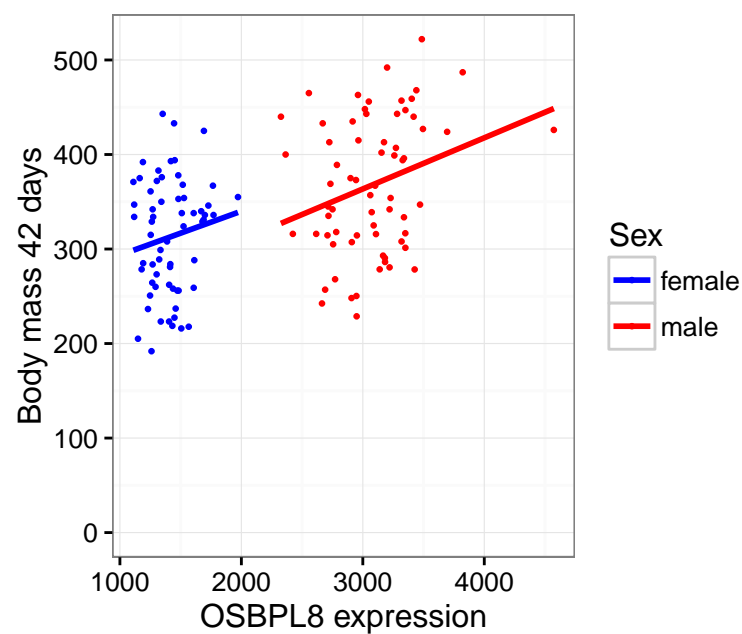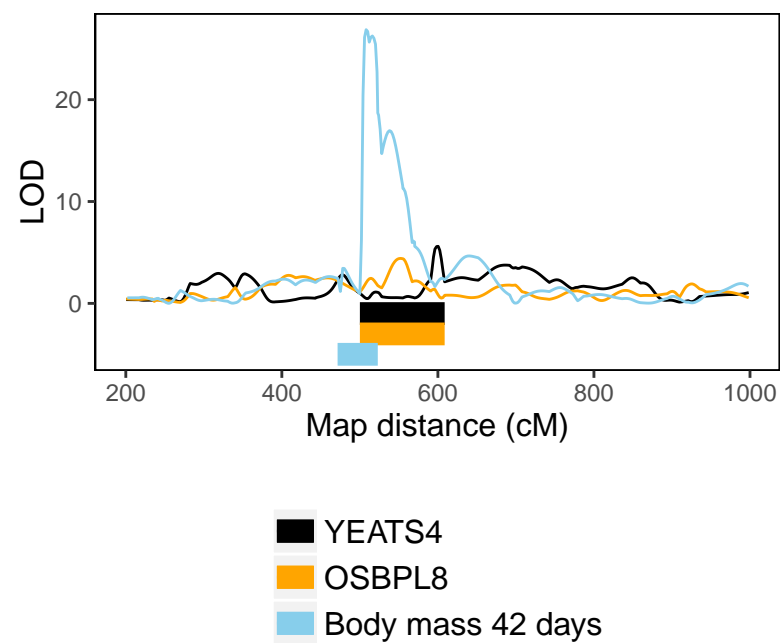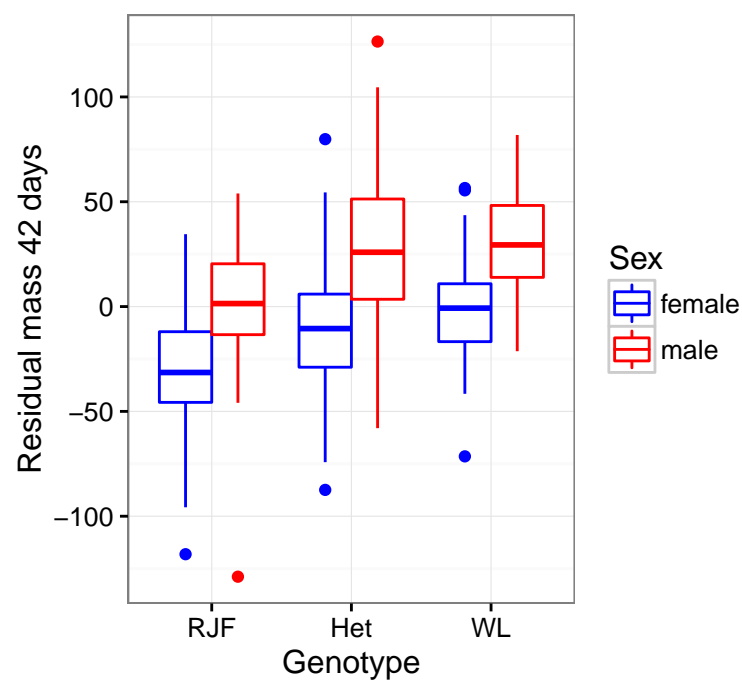

Supplement: Supplementary file 6 — YEATS4 and OSBPL8 candidate plots. (PDF 19 kb) [file 12864_2018_4441_MOESM6_ESM.pdf]

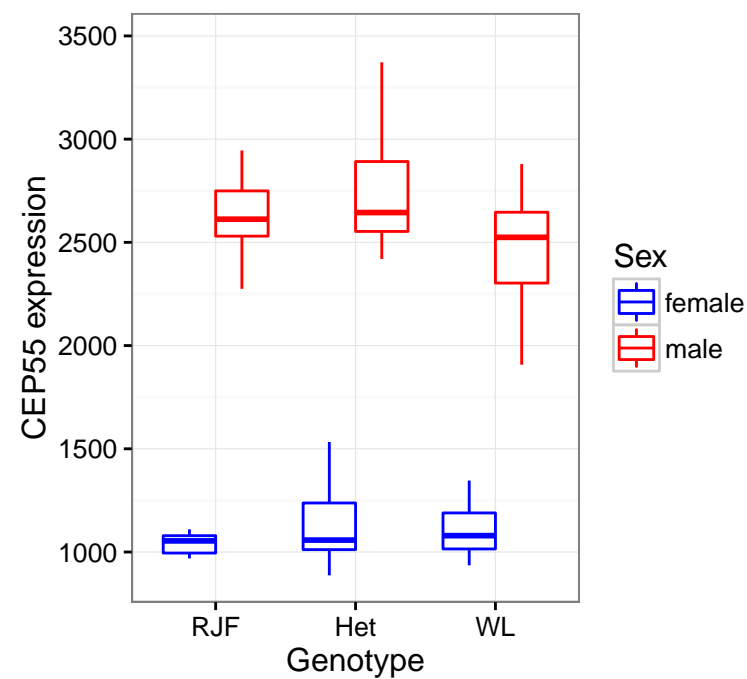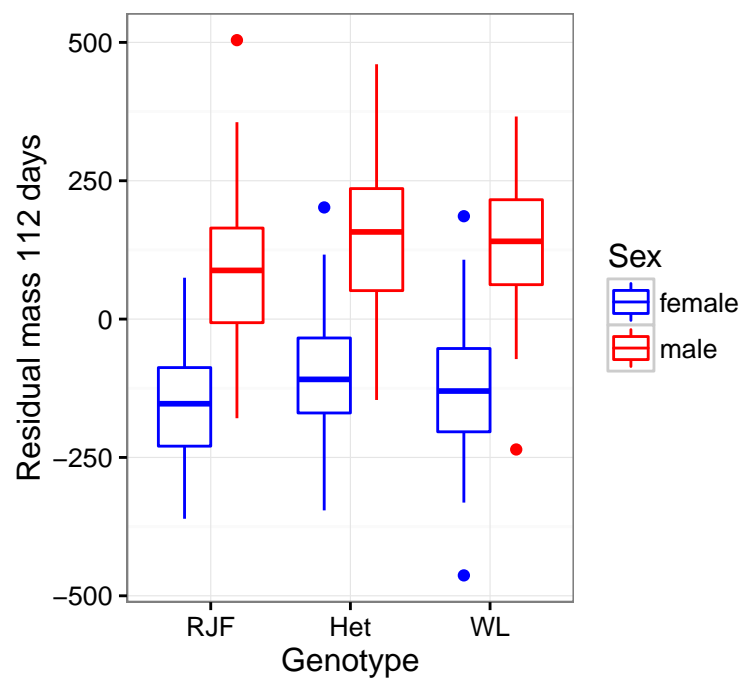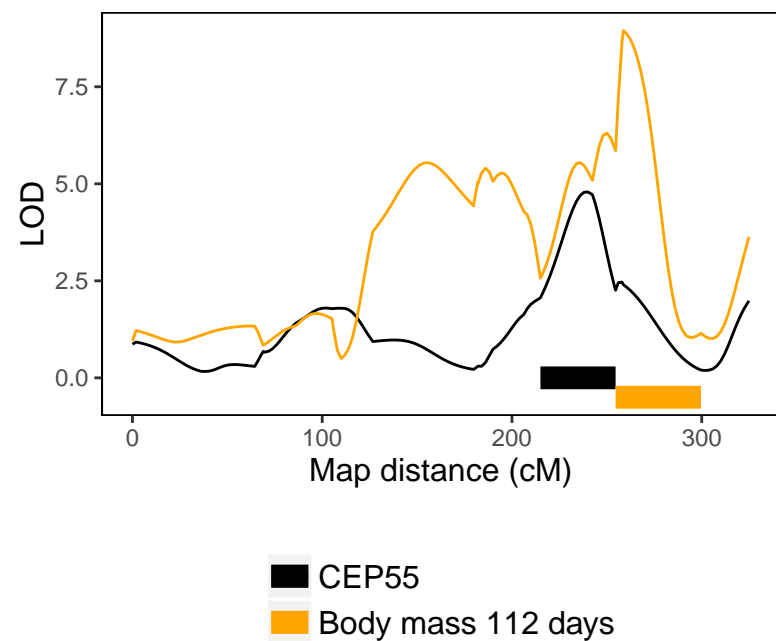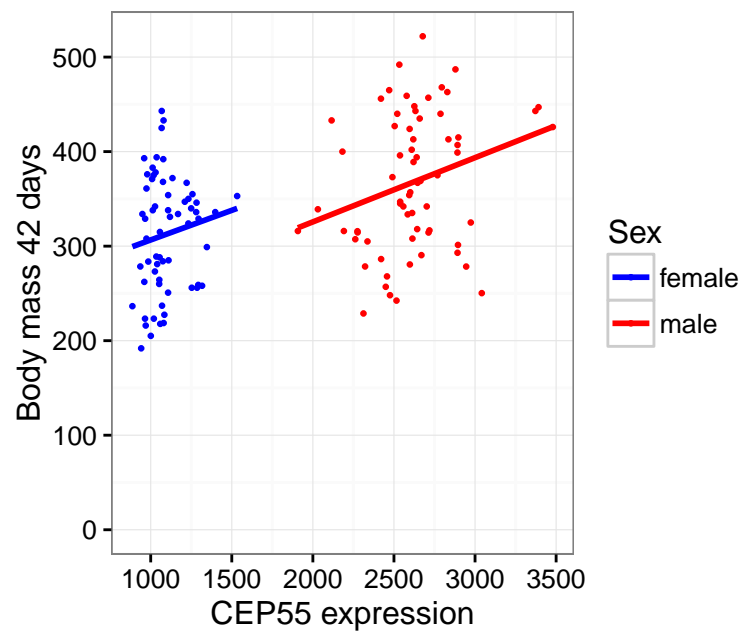

Supplement: Supplementary file 7 — CEP55 candidate plots. (PDF 11 kb) [file 12864_2018_4441_MOESM7_ESM.pdf]

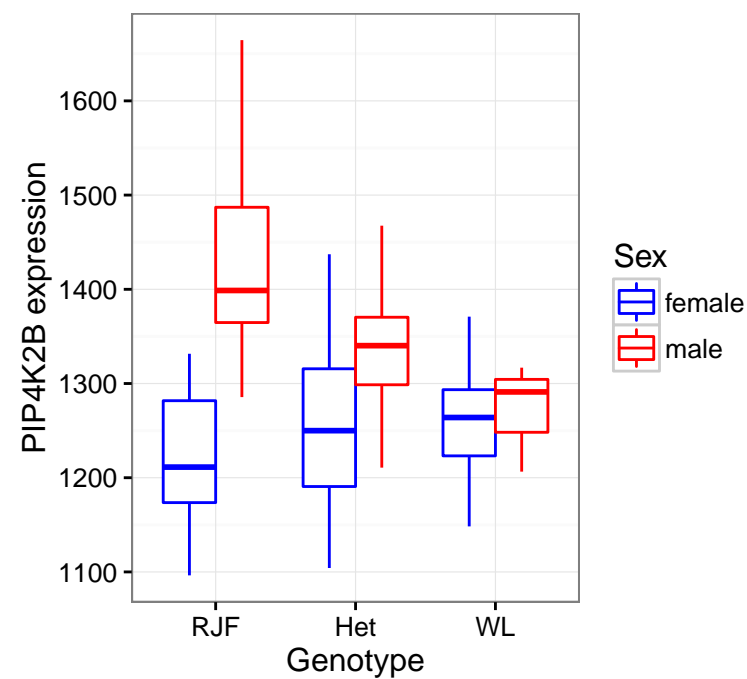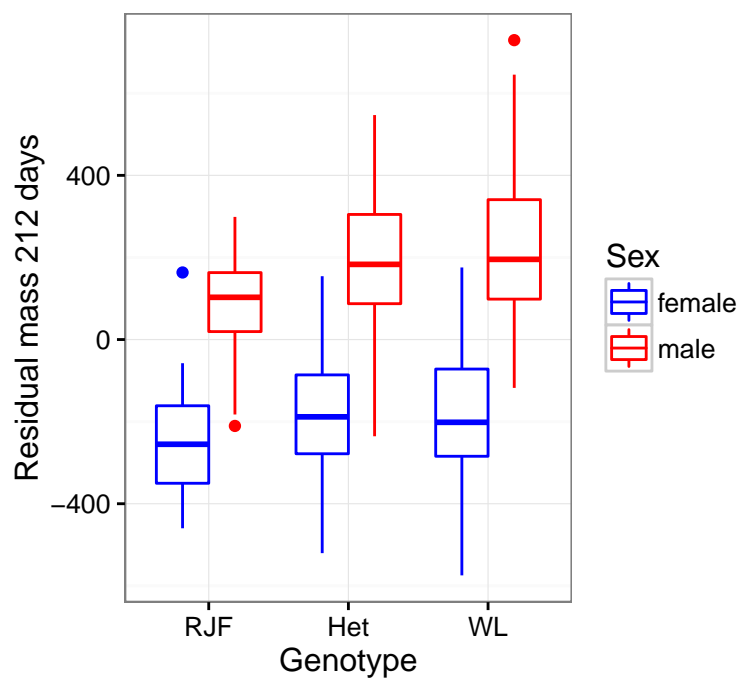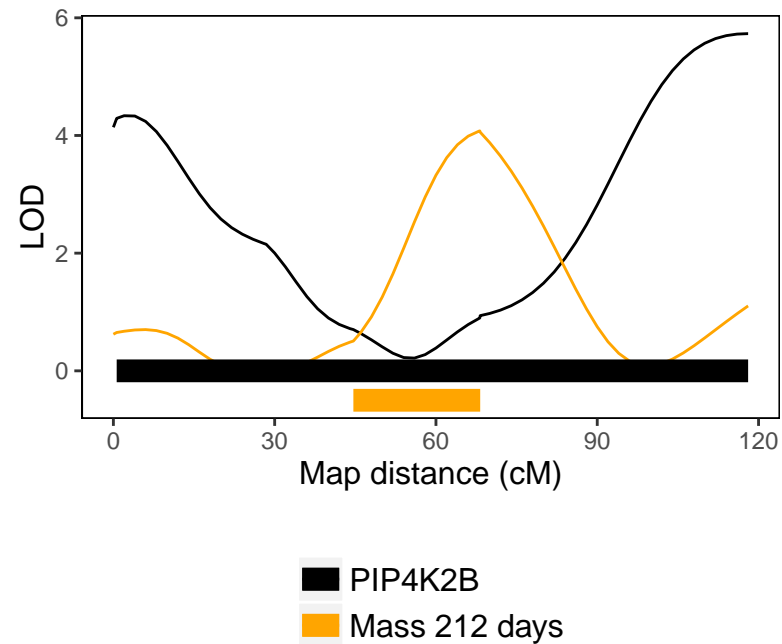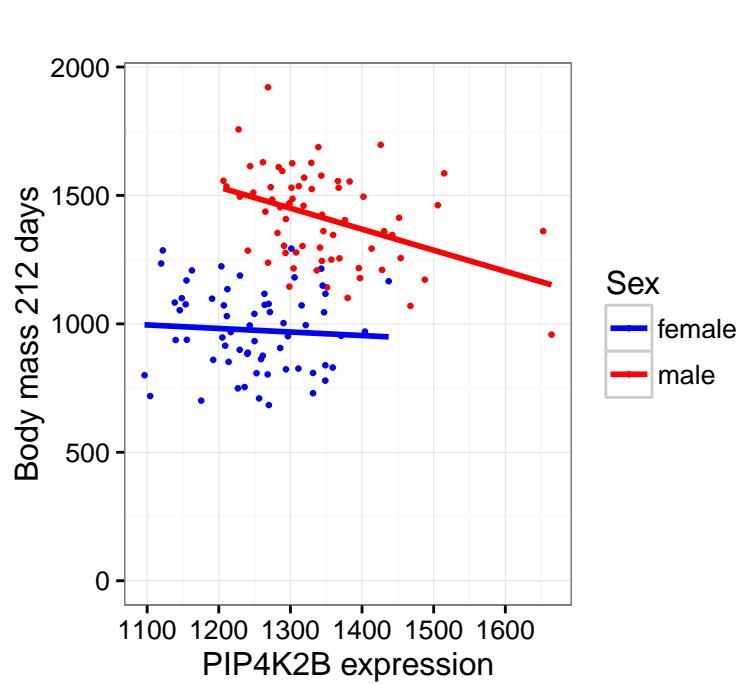

Supplement: Supplementary file 8 — PIP42KB candidate plots. (PDF 10 kb) [file 12864_2018_4441_MOESM8_ESM.pdf]

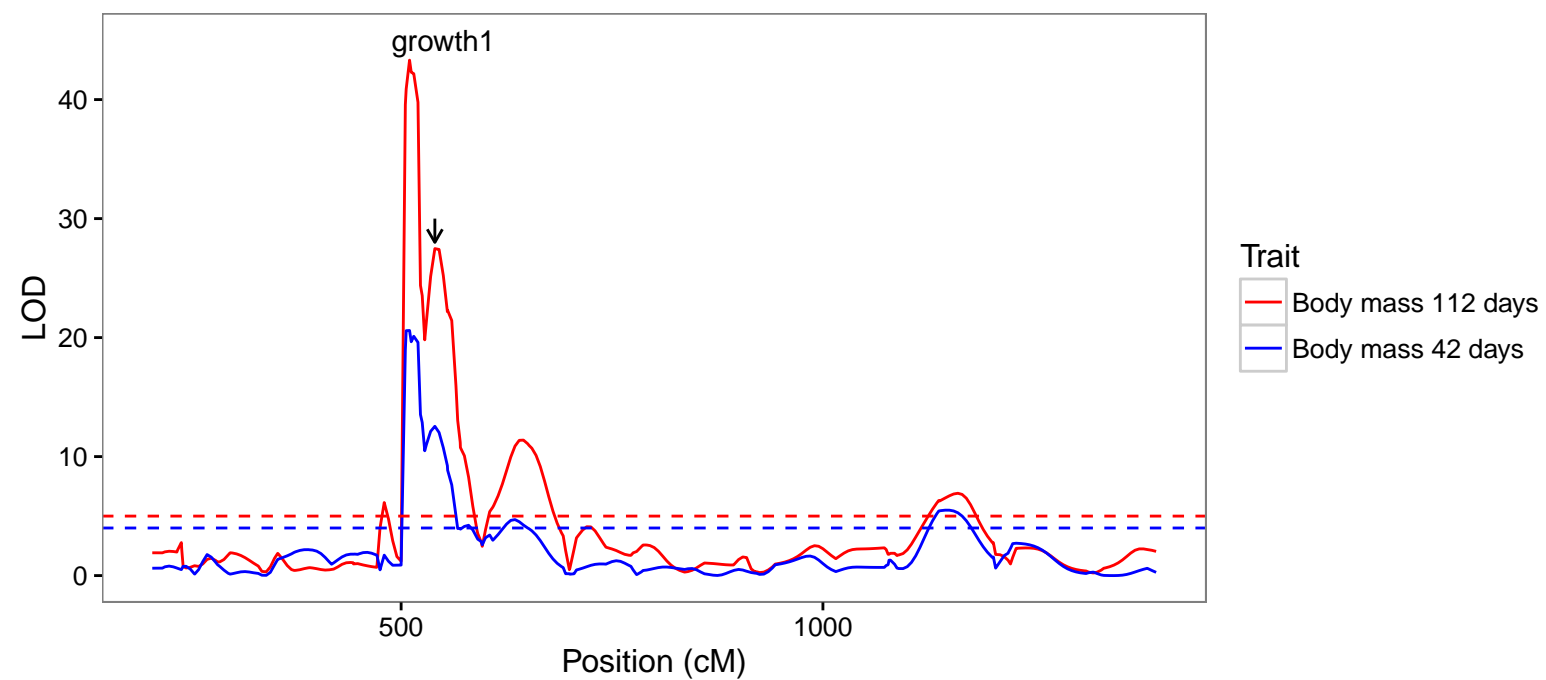

Supplement: Supplementary file 9 — Logarithm of odds curves for the region of chromosome 1 containing the two significant QTL, and a potential second peak, after growth1, which may be a second, imperfectly resolved QTL. The dashed lines indicate genome-wide significance thresholds for the respective trait. (PDF 7 kb) [file 12864_2018_4441_MOESM9_ESM.pdf]
