# Supplementary material for: Genetical genomics of growth in a chicken model
Source: BMC Genomics. 2018 Jan 23;19:72. doi: 10.1186/s12864-018-4441-3 (PMC5782384; doi:10.1186/s12864-018-4441-3)
Supplement: Supplementary file 11 — Plots of gene expression versus genotype for eQTL of the chromosome 5 hotspot. (PDF 22 kb) [file 12864_2018_4441_MOESM11_ESM.pdf]

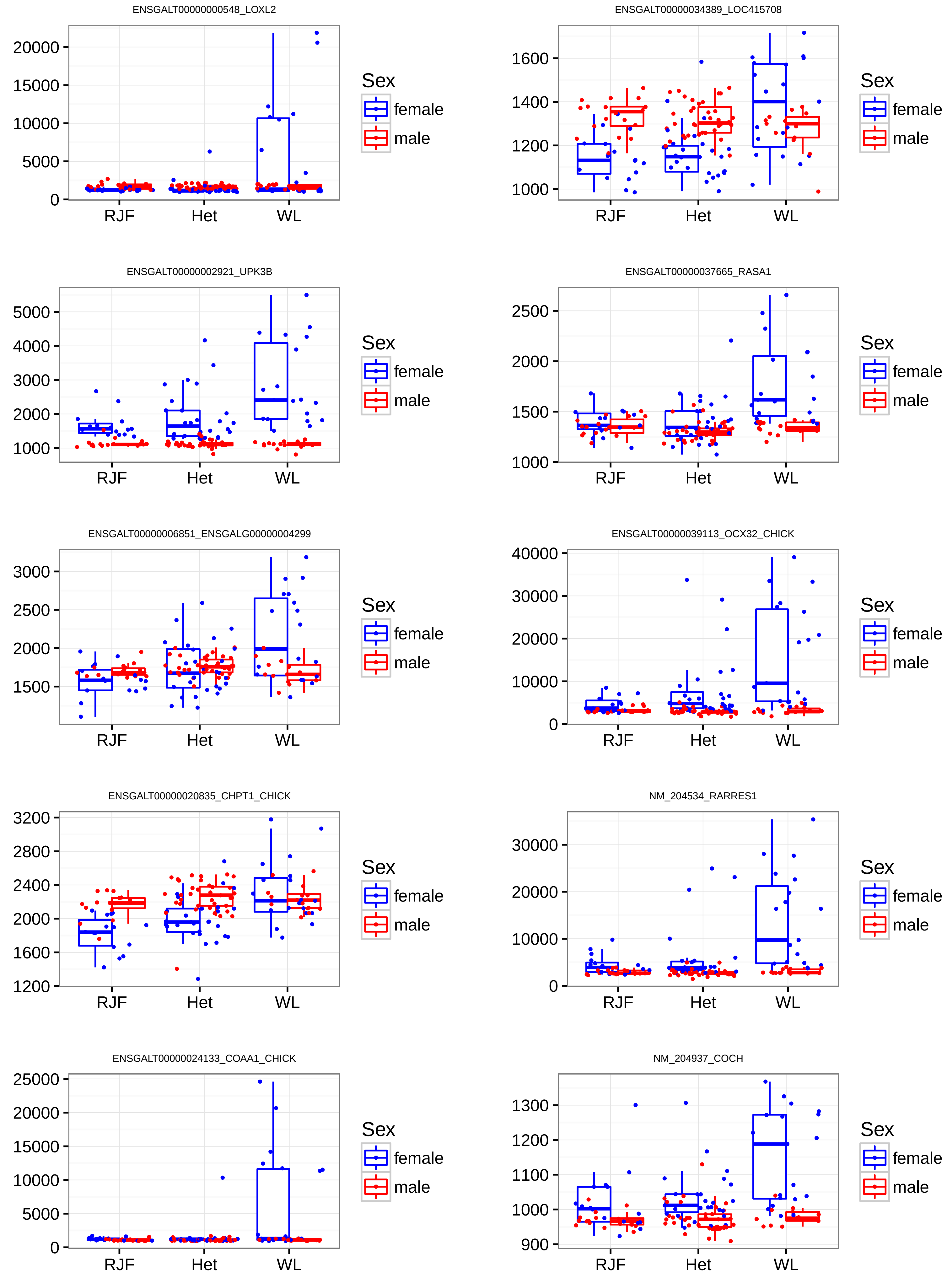
ENSGALT00000000548_LOXL2
ENSGALT00000034389_LOC415708
ENSGALT00000002921_UPK3B
ENSGALT00000037665_RASA1
ENSGALT00000006851_ENSGALG00000004299
ENSGALT00000039113_OCX32_CHICK
ENSGALT00000020835_CHPT1_CHICK
NM_204534_RARRES1
ENSGALT00000024133_COAA1_CHICK
NM_204937_COCH
RJF
Het
WL
Sex
female
male
